# Special Issue: Mucorales and Mucormycosis

**DOI:** 10.3390/jof6010006

**Published:** 2019-12-23

**Authors:** Eric Dannaoui, Michaela Lackner

**Affiliations:** 1 Service de Microbiologie, Unité de Parasitologie-Mycologie, Hôpital Européen Georges Pompidou, F-75015 Paris, France; 2 Faculté de Médecine, Université Paris Descartes, F-75006 Paris, France; 3 Institute of Hygiene and Medical Microbiology, Medical University of Innsbruck (MUI), Schöpfstrasse 41, 6020 Innsbruck, Austria

## 1. Introduction

Mucormycosis is a life-threatening infection, occurring mainly in immunocompromised patients, but also in immunocompetent patients after traumatic injuries [1]. The infection is difficult to diagnose and to treat [2]. The fungi responsible for this disease, the Mucorales, are evolutionary diverse, belong to several distantly related genera [3] and are characterized by their intrinsic resistance to short-tailed azoles [4] and the whole echinocandin substance class [5]. A better knowledge of the biology of mucormycetes and the disease entities caused by them is of prime importance for an accurate and fast diagnosis [6,7,8] and subsequent targeted treatment strategy [9] for patients suffering from mucormycosis.

In this Special Issue, we aimed to highlight and summarize novel aspects for Mucorales and mucormycosis, covering the fields of phylogeny, ecology, epidemiology, diagnosis, disease entities and animal models, thus enabling us to study pathogenesis and novel drugs.

Prakash et al. [10] provide insight into the epidemiology of mucormycosis at a global scale. In particular, changes in epidemiology of the disease are described, highlighting new patient cohorts at risk, namely, patients with a history of tuberculosis and patients suffering from chronic renal failure. In immunocompetent patients, new clinical entities are renal mucormycosis and indolent mucormycosis of nasal sinuses. Causative agents were found to be highly depending on geographical location. *Apophysomyces variabilis* has its highest prevalence in Asia and *Lichtheimia* spp. in Europe. A very detailed estimation of mucormycosis burden in different countries is provided.

Clinical presentation of mucormycosis may be very diverse. Serris et al. [11] reviewed the different forms of the disease and detailed both the clinical and radiological characteristics of the rhino–orbito–cerebral, pulmonary, cutaneous, gastro-intestinal and disseminated mucormycosis. A chapter also explores the healthcare-associated mucormycosis that could occur in newborns, in patients with hematological disorders or transplantation, as well as in burn patients who are particularly susceptible to fungal infections due to the alteration of the skin barrier.

The importance of cutaneous mucormycosis is highlighted in the paper of Devauchelle et al. [12] who performed a systematic review of all cases of mucormycosis in burn patients reported in the international literature from 1990. The authors carefully described the prevalence, the epidemiological and clinical characteristics, as well as the diagnostic and therapeutic challenges of this clinical entity. While the incidence (<1%) is relatively low, the mortality remains high. Soil exposure is often reported in individual cases and in some instances nosocomial outbreaks may occur. The new diagnostic tools (qPCR) are discussed and showed promising results.

Beside burns, necrotizing wound mucormycosis may also be seen in the context of natural disasters, civilian trauma and military injuries. Walsh et al. [13] reviewed the different aspects (from epidemiology to treatment) of wound-associated mucormycosis. The authors showed that the pathogenesis of these infections, although not well known, depends on the traumatic inoculation of foreign materials contaminated with soil-borne fungal elements and the trauma-associated host factors. These infections may occur following combat-related injuries, but also after agricultural or traffic accidents in civilians. Victims of natural disasters are also at risk for wound mucormycosis as shown in patients sustaining trauma during the 2004 Indian Ocean tsunami and the 2011 Missouri tornado.

Animal models are important both to understand the pathogenicity and virulence of fungal agents, as well as to test new therapeutic strategies. This is particularly true for rare diseases such as mucormycosis for which clinical trials are very difficult to set-up. In a comprehensive review about animal models in mucormycosis, Jacobsen [14] showed that different mammalian models (mainly in mice and rabbits) of pulmonary and disseminated mucormycosis have been used to analyze the pathogenesis of the disease, to compare the virulence of the different species and to evaluate the efficacy of different treatments. Nevertheless, although mammalian models have several advantages, ethical and practical considerations limit their use. For these reasons, alternative vertebrate models such as zebrafish or embryonated chicken eggs have been used with success. Invertebrate models such as *Galleria mellonella* or *Drosophila melanogaster* are also increasingly employed for virulence and therapeutic studies.

The portals of entry for mucormycetes are (a) the respiratory tract, (b) the skin and (c) the gut, corresponding to the three exposure pathways: inhalation, direct contact or ingestion, respectively. According to these exposure pathways, different niches and reservoirs are discussed by Richardson et al. [15]. A detailed overview on spore dispersal is given, explaining the different distribution mechanisms in the air. In general, sporangiospores vary between 3 and 11 µm in diameter and are easily aerosolized [16]. The environmental sources for skin infections are comprehensively reviewed, highlighting sources such as soil, plant debris, water bodies, dung and organic materials. Cutaneous infections are mainly linked with traumatic inoculations or contamination of wounds. Novel is that insects, such as flies and spiders, can also carry spores and potentially can transmit them [17,18], as well as that mucormycetes are also prevalent in sediments (bottom soils) of the White Sea at depths of 10–30 m [19]. Mucormycetes can even grow in sea water at low temperatures and varying oxygen levels [19]. Dung of herbivores (sheep and cattle) also provide a reservoir for a wide diversity of mucormycetes. Consumption of mucormycete-contaminated food items (e.g., fruits, dairy products, cheese and yogurt) or mucormycete-fermented food items (e.g., soya sauce) might represent a potential risk for immunocompromised patients for the development of gastrointestinal mucormycosis.

The order Mucorales comprises 260 accepted species in 55 genera. Out of these, 38 were reported from human infections. The molecular revision of the order resulted in numerous taxonomical changes [3]. The Mucorales were assigned to the phylum Mucoromycota. Spatafora et al. [20] conducted genome studies of 46 taxa and found that the zygomycetes comprised two clades with a paraphyletic relationship, namely the Mucoromycota and the Zoopagomycota. The majority of the human-pathogenic fungi are found within the Mucorales. For species identification of the Mucorales in cultures, ITS sequencing remains the gold standard.

In clinics, diagnosis of mucormycosis is based on culture and direct detection of the causative agent in patient material (biopsy, bronchoalveolar lavage (BALs), blood, serum, plasma, urine) by molecular methods. In critically ill patients, non-invasive samples, such as blood, plasma or serum, have been successfully evaluated for diagnosing mucormycosis [8]. For tissue samples, the major conclusion, as highlighted by the European Society of Clinical Microbiology and Infectious Diseases (ESCMID) Fungal Infection Study Group (EFISG) and the European Confederation of Medical Mycology (ECMM) joint clinical guidelines [21], was that fresh/frozen samples performed better than FFPE samples, independent of the PCR format (a PCR with panfungal primers or Mucorales-specific primers, or a combination of specific quantitative PCRs) used. For BALs, Springer et al. suggested the usage of both the cell pellet and the supernatant to improve the sensitivity of the technique [22]. Blood, serum and plasma were investigated as non-invasive alternatives for mucormycosis screening. False positives are rare and only occurs in the very early diagnosis when very low DNA quantities are detected (Cq > 41); thus, a verification by a second sample should be requested. Mucorales DNA load in serum of patients suffering from mucormycosis was high, but still a minimum of 1 mL of sample volume is suggested to ensure high sensitivity [23]. False positives with the Mucorales qPCR blood-based assays are very rare if stringent precautions against contamination are taken, but they may happen. A novel universal biomarker for the detection of mucormycosis was evaluated on urine samples [24]. The marker is a gene family of spore coating encoding proteins (CotH) that exclusively is found in mucormycetes. First results from animal models are promising, but the marker should be validated on a larger number of patients.

Identification of Mucorales to the species level is notoriously difficult by standard morphological techniques. Although the molecular identification of these fungi is now well standardized, less time-consuming and faster techniques are needed. In that respect, Matrix-Assisted Laser Desorption Ionization Time-of-Flight Mass Spectrometry (MALDI-TOF MS) has been a valuable technical advance for the precise identification of yeasts in routine clinical laboratories. Identification of filamentous fungi by this technique is still in progress. Schwarz et al. [25] constructed and validated a database for Mucorales and showed that MALDI-TOF MS is a reliable and rapid method for the identification of most of the human pathogenic Mucorales to the species level.

From a therapeutic point, mucormycosis represents a challenge and alternative therapeutic agents and treatment strategies are desperately needed to improve patients’ outcome. Currently, three agents are available for systemic treatment of deep-seeded infection: amphotericin B, posaconazole and isavuconazole. Among the new strategies, combination therapy is an interesting approach. Macedo et al. [26] have evaluated voriconazole-containing combinations bot in vitro and in vivo. By testing 25 strains of six different Mucorales species, the authors showed that the in vitro interaction of voriconazole with either amphotericin B or caspofungin was mostly indifferent. In contrast, they showed that these two combinations were beneficial in vivo against *Rhizopus microsporus* in a *G. mellonella* model. Further studies are needed to confirm these results. 

## 2. Conclusions and Outlook

The taxonomic position of Mucorales has been solved and a greater understanding of the high phylogenetic levels, such as orders and families, achieved, but still a lot of uncertainties and discussions exist on the genus level and below. Understanding the biology of mucormycetes is key to understand human infections and infectious sources. In the past years, novel niches and reservoirs have been identified for a better understanding of potential exposure sources. However, comprehensive data on indoor and outdoor spore concentrations are missing. Even though several vertebrate and invertebrate models were developed for invasive mucormycosis, the minimum infectious doses via a natural pathway of entry are not understood. Further studies are needed to understand how infections can be acquired from the environment. However, early diagnosis and issues around accurate pathogen identification has been solved. Novel molecular methods have been established for all sorts of clinical specimens; the next step is standardizing and comprehensively evaluating the methods at hand. Improved early diagnostics is a major advance towards efficient management of mucormycosis by the better identification of cultured isolates, but also by direct identification on clinical tissue samples.
